# Environmental DNA as a tool to reconstruct catch composition for longline fisheries vessels

**DOI:** 10.1038/s41598-024-60917-7

**Published:** 2024-05-03

**Authors:** M. E. Green, B. D. Hardesty, B. E. Deagle, C. Wilcox

**Affiliations:** 1 Institute for Marine and Antactic Studies, University of Tasmania, Private Bag 49, Hobart, TAS 7001 Australia; 2 Centre for Marine Socioecology, University of Tasmania, Private Bag 49, Hobart, TAS 7001 Australia; 3 CSIRO Environment, Castray Esplanade, Hobart, TAS 7001 Australia; 4 CSIRO Australian National Fish Collection, Castray Esplanade, Hobart, TAS 7001 Australia; 5 Wilco Analytics, 93 Carlton Beach Road, Dodges Ferry, TAS 7173 Australia

**Keywords:** Marine fisheries, Longlining, DNA-metabarcoding, eDNA, PCR-based techniques, Marine biology

## Abstract

Global wild-capture fisheries are a large and diverse sector requiring various tools for fisheries-dependant data collection and effective Monitoring, Control and Surveillance (MCS). Here we present a novel protocol to collect eDNA from brine tanks onboard commercial longline vessels to reconstruct catch composition. We collected samples from nine vessels operating out of the Eastern Tuna Billfish Fishery, Australia, validating eDNA results with reliable catch data consisting of seven target and bycatch species. Environmental DNA was highly effective for detecting species retained on vessels without contamination or false positives. For four vessels, logbook data and eDNA were consistent with detections of all species. The remaining vessels detected all species except for rare catches of short-billed spearfish (*Tetrapturus angustirostris*). Similarities between rank abundance distributions of catch and eDNA reads were observed with logbook data mirrored when eDNA sequences were organised into rank order abundance. The method was effective at identifying highly abundant taxa retained in brine tanks- tuna (*Thunnus spp.),* swordfish (*Xiphias gladius)*, marlin (*Kajijia audax)*, and Atlantic Pomfret (*Brama brama*). Further research is required to validate how eDNA and other molecular monitoring tools can be scaled and applied to provide solutions for monitoring challenges in the fisheries sector.

## Introduction

In 2020 global capture fisheries produced 90.3 million tonnes, valued at $141 billion USD^1^. Seafood consumption has risen steadily by 3 per cent annually since 1961^1^ fuelled by increased access to more distant markets^2^. Seafood comprises 49% of all internationally traded meats significantly surpassing bovine (19%) and poultry (11%) trade^3^. The wild-caught seafood industry sustains over 600 million livelihoods and serves as a critical revenue source and employer for many countries^1^, impacting both domestic and global economies, and marine ecosystems profoundly. Successfully managing fishing activity is a ‘wicked challenge’ given the scale and diversity of the sector^4^. Consequently, the catch composition and activity of many fisheries are not monitored or are uncertain (e.g., data-limited) and some fishing activity can be defined as Illegal, Unreported and Unregulated (IUU). Data-limited fish species make up more than 80% of global fisheries stocks, lacking adequate data for formal stock assessments^5^. This shortcoming is frequently compounded by a limited understanding of catch, landings and stock dynamics^6^, with management agencies often having reduced capacity for effective management^7^. IUU fishing is known to be harmful to fish stocks and economies^8^. The most recent estimate is that the annual global IUU catch is between 11–26 million tons with a value of USD $10-$23.5 billion in losses^9^. Both data-limited fisheries and IUU activity can result in a lack of adequate knowledge of what fish are being caught where and by whom, making it difficult to maintain the sustainability of fish stocks, ecosystems, and livelihood^6,10^. Currently, 4.5 million tons of reported catch is considered unidentified^1^, this figure does not include additional IUU catch (11–26 million tons per year)^9^.

These figures highlight the considerable opportunity present to enhance our global understanding and resolution of fisheries catch. Current methods for monitoring fishing activity include logbooks, electronic monitoring (EM), on-board observers and port-side or high-seas inspections^11,12^. Each of these methods, though effective tools for Monitoring, Control and Surveillance (MCS) of fishing, are not all accessible to some fisheries (especially EM) and have weak points in the process where non-compliant behaviour can go unmonitored. For example, not all fisheries, fleets or vessels are observed at-sea or inspected on arrival in port^13^; catch composition reporting in logbooks can be incorrect or falsified^14^; observer reporting and records may be biased^15^; not all fisheries, fleets and vessels have the capacity and resources to install and maintain EM^12^. Moreover, if a vessel inspection is undertaken, the complexity of identifying lookalike species (e.g. tuna, whiting, marlin and sharks) once they are processed make it challenging to identify species accurately. This issue is clearly visible in the UN FAO database, the world’s largest dataset on seafood capture and landings; 40% of all wild-caught landings are not reported to species level^16^.

Molecular monitoring (MM) is a tool that uses DNA techniques and other molecular-based approaches to better understand seafood supply chains where regular monitoring and surveillance is limited, challenging or unavailable^17^. DNA can be extracted from numerous sources to taxonomically identify the species present. The application of MM across fisheries is broad and includes detecting illegal trade of species in fish markets^18–20^, food products^21^, pet foods and beauty products^22^, identifying mislabelling in human food products (i.e., canned tuna, processed fillets)^23–25^, detecting harmful bacteria in raw seafood^26^ and monitoring the trade of commercially important species such as tuna and salmon^27–32^.

Recently, there has been academic interest in using environmental DNA to assist with fisheries monitoring and reporting^33–37^. Environmental DNA or eDNA is a mixture of genomic DNA from different organisms found in samples such as water, soil, air, and faecal matter^38^. Advances in extraction methods and sequencing technologies have made it possible for eDNA methods to successfully identify many species in a variety of environments^39,40^. Recent research has showcased the feasibility of collecting eDNA from commercial fishing vessels including the brine tanks where catch is stored^41^ and while trawl nets are being towed in the water column^36,37,42^. These studies demonstrate that eDNA can be collected in such environments and DNA from some target species is present in samples. However, the relationship between eDNA detections and true catch is yet to be validated. In previous studies, eDNA collected in brine tanks was unable to be compared with logbook data meaning the true catch retained in tanks was unknown^41^. While eDNA sampling undertaken in trawl nets during trawling activities detects species from outside of the trawl nets, reducing the reliability of the methods for catch assessments^37,42^.

The aim of this study was to develop a method to sample brine tanks onboard fishing vessels and reconstruct landed species using a molecular monitoring (MM) approach. Samples were collected from tanks with known quantities of fish to validate eDNA results and determine how effective eDNA is as a monitoring tool in this context. Chilled seawater systems, brine tanks and ice slurry systems are commonly used in commercial fisheries to preserve the freshness and quality of catch^43^. The tanks are often seen on vessels fishing for tuna or salmon, or pelagic and longline vessels, and brine tanks are sometimes used in groundfish fisheries. The conditions in a brine tank (i.e., low light, cold temperatures and abundance of source DNA) offer an excellent opportunity for preserving eDNA.

## Material and methods

Water samples were collected from 9 commercial longline fishing vessels operating out of the Eastern Tuna and Billfish Fishery (ETBF), Mooloolaba, Australia, over two trips; Jan 2021, Mar 2022. Brine tanks on these vessels are filled with seawater and chilled while the longline shot is being deployed, and used to store catch for up to two weeks. The number of brine tanks per vessel varied from two to nine. Weight, species and type of processing was recorded for all catch.

Before eDNA sampling, all required equipment including storage bins were decontaminated with 20% bleach for at least 20 min before being rinsed with purified Milli-Q® water (Millipore, USA). To collect eDNA from brine tank samples, water samples were collected using a 1L wide mouth Nalgene bottle held in the brine tank with a gloved hand. For ambient temperature storage of eDNA a precipitate method was used, whereby a small volume of water is preserved in DNA-free Longmires solution^44,45^. This involved mixing 15 mL of brine tank sample (decanted from the 1L Nalgene bottle) and 5 mL of Longmires solution into a 50 mL Lo-Bind falcon tube (Eppendorf, Germany). A total of 10 sample replicates were collected from each tank on each vessel. The number of brine tanks per vessel and samples used for analysis are described in Supplementary Table S1. The ratio of Longmires solution to sample (i.e. 1:3) was selected as it has been previously demonstrated as successful for preserving eDNA^45,46^. Whole samples were stored in the dark, at ambient temperatures and transported to CSIRO Marine Laboratories, Hobart, Australia.

Whole samples preserved in Longmire’s solution had eDNA extracted using a precipitation method (PPLPP) as described in Edmunds and Burrows (2020)^47^ and purified using DNeasy PowerClean Pro Clean up Kit (Qiagen, Germany) following manufacturer’s instructions. eDNA was eluted into a final volume of 100 µL. In order to detect any cross-contamination, negative controls containing only Milli-Q® water (Millipore, USA) were extracted and processed alongside all samples.

Extracted eDNA was sent to eDNA Frontiers, Curtain University, Western Australia for DNA metabarcoding of the mitochondrial 16S region using universal fish primers; Fwd:GACCCTATGGAGCTTTAGAC^48^ and Rev:CGCTGTTATCCCTADRGTAACT^49^. These primers amplify a ~ 200 bp amplicon and are widely applied for fish identification in eDNA studies^50,51^. To determine the required dilution for optimal amplification and assess if inhibition would affect reactions, initial trial PCR reactions were performed with neat, 1/10 and 1/100 dilutions of DNA. The PCRs were performed at a final volume of 25µL where each reaction comprised of: 1 × PCR Gold Buffer (Applied Biosystems), 0.25 mM dNTP mix (Astral Scientific, Australia), 2 mM MgCl_2_ (Applied Biosystems), 1U AmpliTaq Gold DNA polymerase (Applied Biosystems), 0.4 mg/mL bovine serum albumin (Fisher Biotec), 0.4 µM forward and reverse primers, 0.6 μl of a 1:10,000 solution of SYBR Green dye (Life Technologies), and 2µL template DNA. PCRs were performed on StepOne Plus instruments (Applied Biosystems) with the following cycling conditions: 95 °C for 5 min, followed by 50 cycles of: 95 °C for 30 s, 54 °C (16S) for 30 s, 72 °C for 45 s, then a melt-curve analysis of: 95 °C for 15 s, 60 °C for 1 min, 95 °C for 15 s, finishing with a final extension stage at 72 °C for 10 min.

After selection of the optimal dilution (neat or 1:10), PCRs were repeated in duplicate as described above but instead using unique, single use combinations of 8 bp multiplex identifier-tagged (MID-tag) primers as described in Koziol et al*.*^52^ and van der Heyde et al*.*^53^. Master mixes were prepared using a QIAgility instrument (Qiagen) in an ultra-clean lab facility, with negative (blank containing MilliQ water) and positive PCR controls (using *Xyrichtys novacula* DNA) included on every plate to ensure the validity of results. A sequencing library was created by combining samples into mini-pools based on the PCR amplification results (delta Rn) from each sample. The mini-pools were then combined in roughly equimolar concentrations to form libraries. Libraries were then size selected (160–450 bp cut-off) using a Pippin Prep instrument (Sage Sciences) with 2% dye-free cassettes, cleaned using a QIAquick PCR purification kit, quantified on a Qubit (Thermo Fisher), and diluted to 2 nM. The libraries were sequenced (single direction) on an Illumina MiSeq instrument using 300-cycle kits with custom sequencing primers.

Bioinformatics were performed using the R Package DADA2 ver. 1.28^54^. Single-direction FASTQ files were demultiplexed and renamed into unique FASTQ files for each marker and sample. All FASTQ files had primers and sequences with ambiguous bases removed. During quality inspection, reads with two or more expected errors (maxEE = 2) were discarded and sequences with minimum quality score (Q) of 30 were preserved. DADA2 was used to denoise sequences, remove chimeras and generate an Amplicon Sequence Variant (ASV) table.

Taxonomic assignment for 16S ASVs was completed using a custom database consisting of families of fish species expected to be in the fishing vessel tank. Each ASV was also queried in NCBI GenBank to ensure the custom database contained all relevant fish species and no closer matches were present in GenBank. The custom database created for this research project is provided in supplementary material (Supplementary File S1). Taxonomy was then assigned using the naïve Bayesian Classifier and function *assignTaxonomy()* in DADA2^54^. Bootstrapping was applied with taxonomic identification considered when the bootstrap values were > 80 (minBoot = 80). These assignments were further validated by manually checking sequences on GenBank using the BLAST function^55^ and confirming species identity when sequence identity was > 98%. At this point ASVs were transformed into Operational Taxonomic Units (OTUs) based on the species assignment results. To reduce the potential for false positives related to tag jumping (Schnell et al. 2015) and/or contamination, low-frequency OTUs within each sample were excluded if the number of reads was < 0.001% per sample.

All statistical analyses and data visualisations were performed in RStudio v2022.12.0 + 353. Catch data including species, abundance, and biomass (weight) were provided by the fisheries operator from their onboard logbooks, which are considered a trusted representation of true catch. The vessels operating in the ETBF are highly managed and regulated with all retained catches recorded in logbooks at sea, during unloading, on catch disposal records and on electronic monitoring cameras set up on vessels.

**Figure 1:**
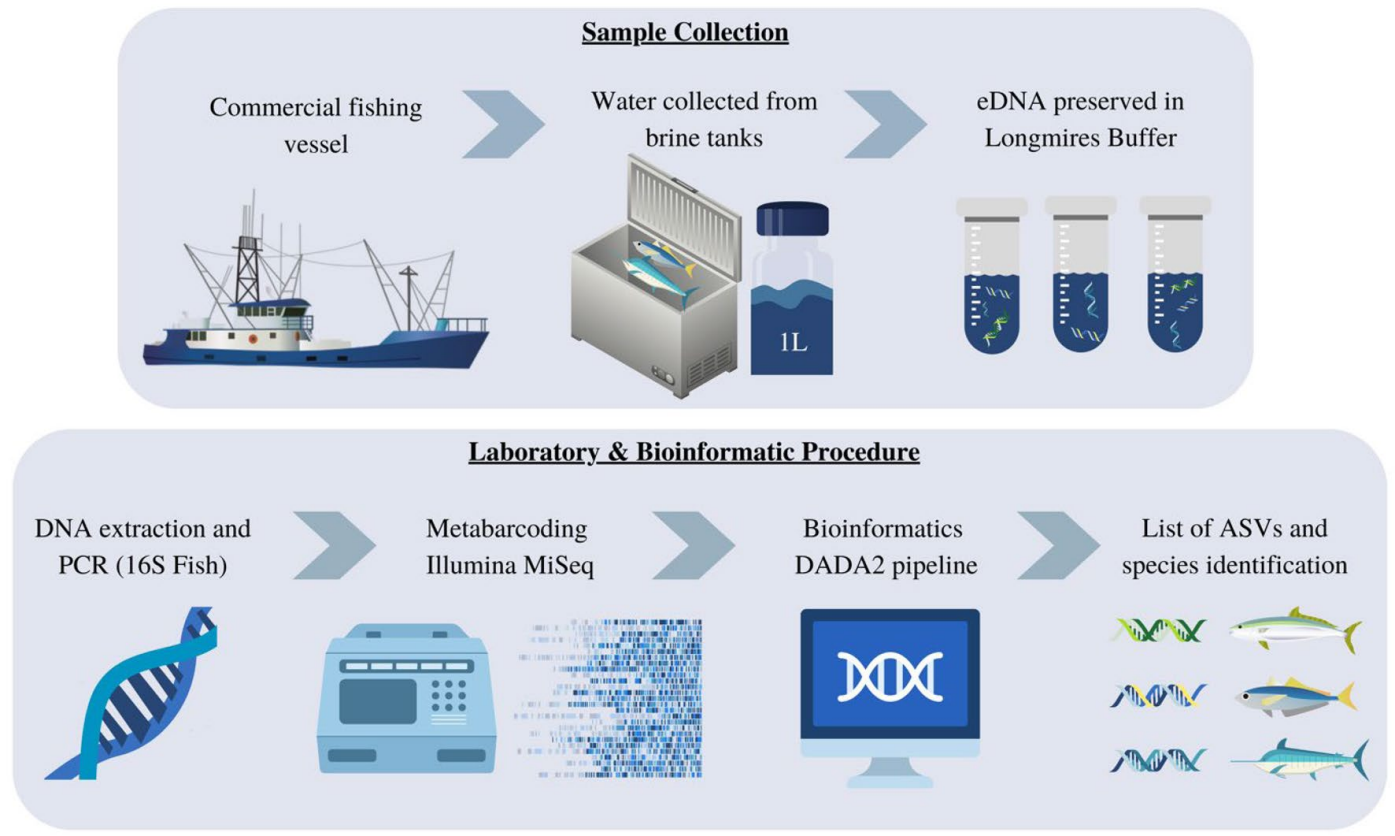
Brine tank sampling for commercial longline vessels. Major components of the process are shown.

To prevent a bias in the molecular data sets between samples due to variable sequencing depths per run, read numbers were transformed into three values. First, sequence reads were converted into a proportion, representing the percentage of each taxon per sample. Second a binary count of detection per taxon, per sample was recorded whereby 1 = present, 0 = absent. Third, to undertake rank abundance distribution testing the total number of reads per taxon per vessel were compared with total catch per vessel (number of individuals). Significant components of the brine tank sampling process and laboratory procedure are shown in Fig. 1.

## Results

A total of 36,074,333 raw reads were generated across 4 sequencing runs. The average raw reads per run was 9,018,583 which equated to a range of 74,477 – 89,443 reads per sample. After quality filtering, taxonomic assignment and discarding contaminated samples, 34,764,345 million reads remained (Table S2). A total of 927 ASVs were identified across 404 samples from nine vessels (Table S3). The DADA2 naïve Bayesian classifier was able to assign all reads to at least the family level (Table S4). Based on DADA2 and BLAST results, ASVs were transformed into 27 OTUs which represented 24 species, one genus and two families of bony fishes. Reads unable to be identified to species level were due to the 16S region being highly conserved, this was especially the case for the billfish family (*Istiophoriformes*) and tuna family and genus (*Scombridae* and *Thunnus spp.*). All reads that could not be assigned to species or the *Thunnus/Katsuwonus* genus at a minimum bootstrap threshold of 80 were not included in downstream analyses. The assignment of tuna ASVs were combined at the genus level (*Thunnus*) as many of the matches in BLAST were > 98% for multiple species. Reads discovered in negative controls were removed by using the subtraction method^56^. Non-target taxa were removed as they did not meet filtering quality thresholds (OTU < 0.001% per sample) or because they were found in a single sample (CA363) which was removed from analysis due to clear contamination (Table S5). Following filtering, the final dataset consisted of 7 OTUs from four species, one genus and two family level assignments (Table S6).

Across the nine vessels, catch data revealed a total of nine species were caught and retained in brine tanks, five species of tuna; Albacore (*Thunnus alalunga*), Big-eye (*T. obesus*), Skipjack (*Katsuwonus pelamis*), Yellowfin (*T. albacares*) and Pacific Bluefin (3 individuals, separate trips) (*T. orientalis*), three species of billfish; Swordfish (*Xiphias gladius*), Striped Marlin (*Kajikia audax*) and Short-billed Spearfish (*Tetrapturus angustirostris*) and one species of bream; Atlantic pomfret (*Brama brama*). The most abundant catch in count and weight was Albacore tuna (*Thunnus alalunga*). While, Short-billed Spearfish (*T. angustirostris*), were the least abundant genus captured with total catch per vessel ranging from 1–7 individuals. As species-level assignment was not possible for eDNA data using the 16S assay for the tuna group, all catch data (of the five species) were combined to represent a single count of abundance and weight for tuna per vessel.

**Figure 2:**
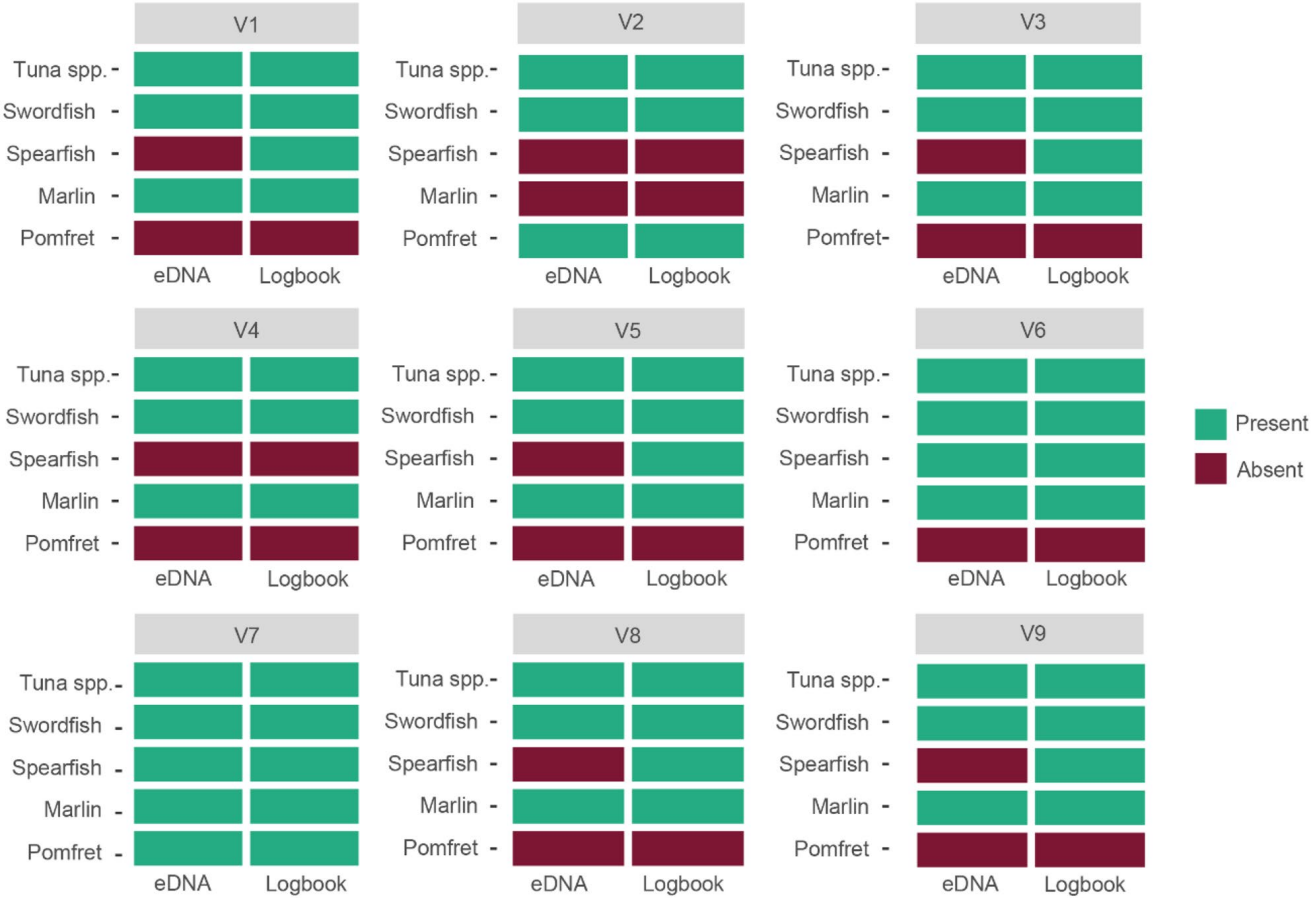
Tile plot indicating presence (green) and absence (red) of species for both eDNA and logbook data per vessel.

All species caught on longline vessels were identified using eDNA and no contamination or false positives occurred in the eDNA dataset (post-filtering). Species recognised in both logbooks and eDNA were Atlantic pomfret (*B. brama*), striped marlin (*K. audax*), short-billed spearfish (*T. angustirostris*), swordfish (*X. gladius*) and tuna as a species complex (labelled *Tuna spp.* herein*).* For vessels 2, 4, 6 and 7 the logbook data and eDNA were 100% consistent, positively identifying all species stored in brine tanks (Fig. 2). The remaining vessels had all species positively identified using eDNA except for spearfish (Fig. 2).

**Figure 3:**
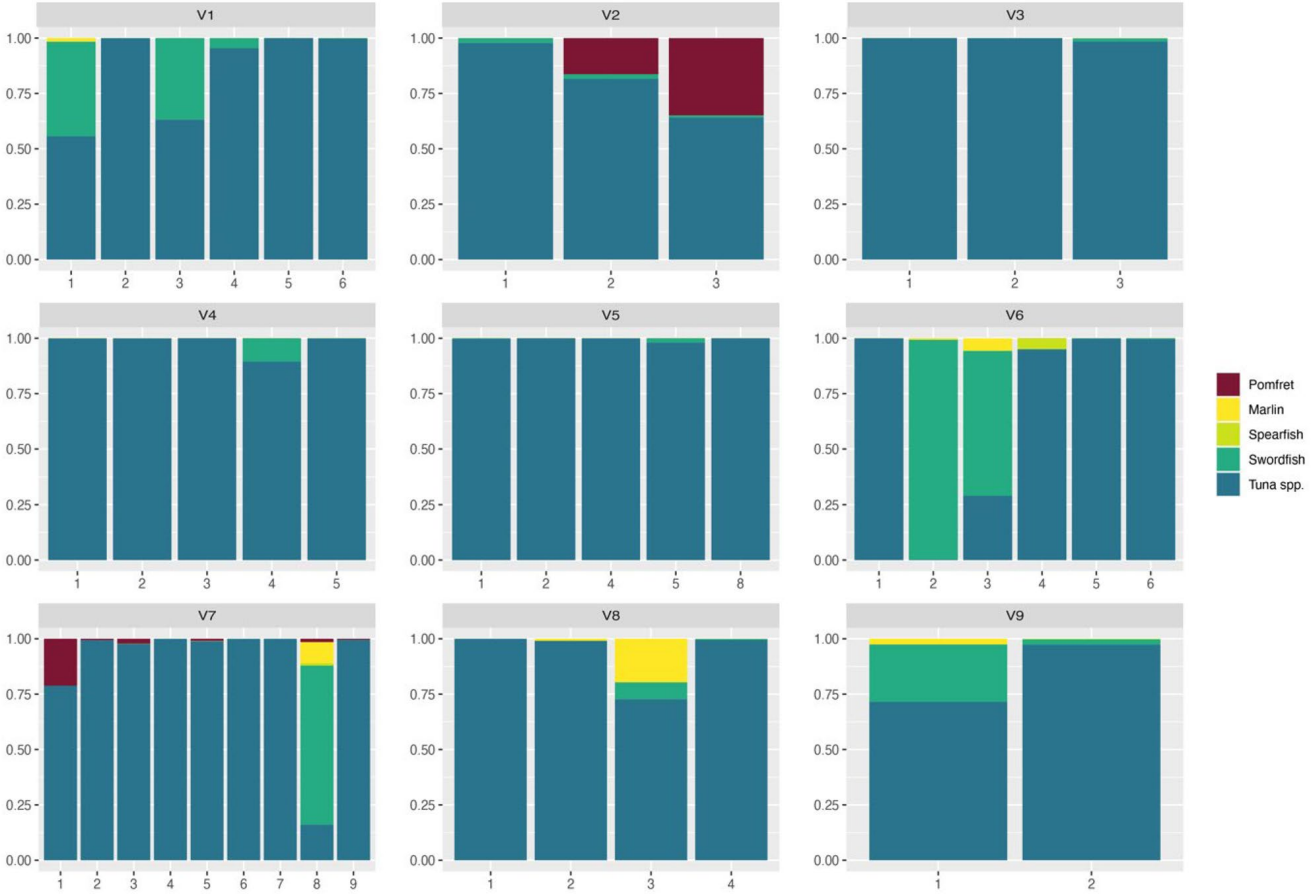
Frequency of species per tank, per vessel. Each bar represents the proportion of eDNA assigned to a species per tank. Number of bars vary as vessel size and number of brine tanks onboard differ between vessels. Sample size per tank ranged from n = 8–10.

Species proportions differed across tanks and the majority of eDNA was identified as tuna for all vessels (Fig. 3). Swordfish were the second most common species in tanks with abundant proportions of reads identified in vessels 1, 6, 7 and 9. For some vessels most billfish and pomfret eDNA reads were identified in a single tank (vessels 4, 7, 8, 9).

**Figure 4:**
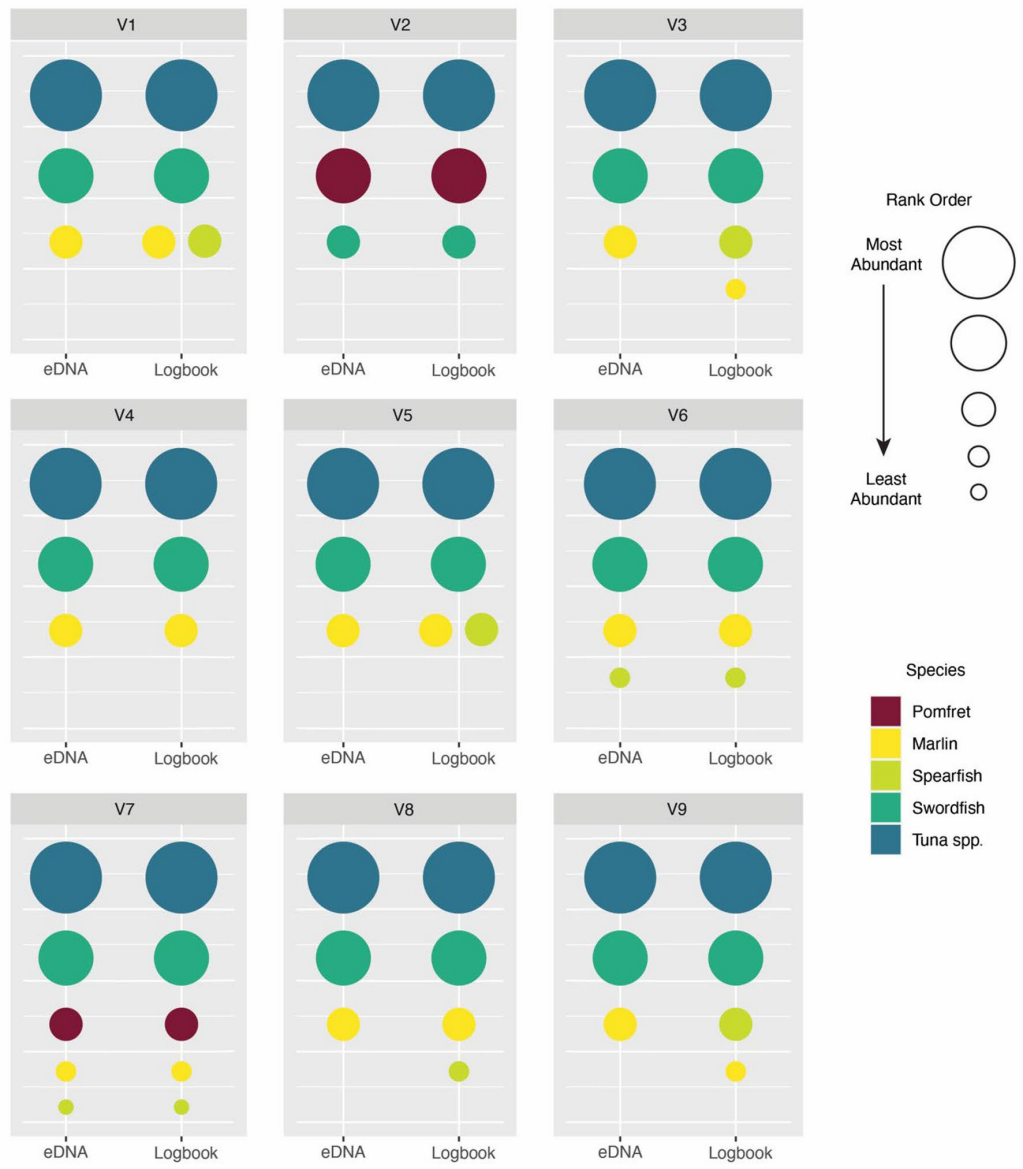
Rank order abundance of eDNA reads (left) and logbook data (right) for each vessel. eDNA reads are the total number of reads identified for each species, per vessel, while logbook data is the representation of total catch in numbers (not weight). Identical catch rank is represented as two circles of the same size (V1 and V5).

The number of eDNA reads and catch abundance were highly similar when organised into rank order abundance distributions (Fig. 4). For all vessels, the two most commonly caught species; tuna and swordfish (or pomfret for vessel 2), were correctly represented in the rank order of eDNA sequences. In the case where all five species were identified using eDNA (vessel 7), the rank of sequences matched the catch abundance as reported in the logbook.

## Discussion

Our results show that eDNA molecular monitoring (MM) is a viable option to authenticate seafood species captured and retained in brine tanks onboard commercial vessels. Environmental DNA successfully identified all species retained in brine tanks with the exception of less abundant catch (short-billed spearfish) for some vessels. Importantly, the method was highly selective with no contamination or detection of species that were not in brine tanks. The method reliably identified highly abundant species retained in brine tanks (tuna-complex, swordfish, marlin and pomfret) and logbook data was effectively mirrored when eDNA sequences were organised into rank order abundance.

The taxonomic assignment of DNA sequences extracted from brine tanks was limited due to the presence of several tuna species that are phylogenetically closely related and therefore show a high similarity between their DNA sequences^57^. Still, we could assign catch with high confidence assignments to groups at family, genus and species level, allowing direct comparison with catch data reported in logbooks. At the genus level, all catch was positively identified by eDNA in all vessels. After stringent bioinformatic filtering and taxonomic assignment, a total of seven taxa were identified across all vessels and no contamination from non-target species (i.e., false positives) was detected. Collecting eDNA from brine tanks onboard commercial vessels does not have the same challenges as standard eDNA studies conducted in the environment because the number of taxa being detected is relatively low and the high abundance of source DNA from retained fish in tanks. Usually, eDNA exists in environments in low concentrations, is often heterogeneously distributed throughout a water body and can be highly variable in space and time^58–60^. We assume the shedding rate of eDNA from fish stored in brine tanks is especially high as all species undergo some level of processing (e.g., headed, gutted, and/or gilled) and are retained in tanks for long periods of time. Tanks are also maintained at cool and stable temperatures and are exposed to low light; conditions known to preserve eDNA well^61^. For many eDNA studies a guiding principle for water sampling is “more is better” to increase the detection probability of rare eDNA signals^60^. However, brine tanks are a relatively small body of water when compared to a river, lake, or open ocean, meaning the concentration of eDNA is likely to be higher. Our results suggest small water volume samples collected from brine tanks are selective enough to collect highly abundant eDNA from processed catch retained in the tanks, however are not sensitive enough to detect trace eDNA which would be present in the seawater that filled the tanks.

Failure to detect species did occur in our dataset but only for catches of short-billed spearfish (five out of the seven vessels that landed spearfish). Several possibilities may explain why spearfish DNA was not detected in brine tank samples. As there were very low numbers of spearfish caught and retained on each vessel the concentration of source eDNA may have been reduced. The small volume samples that have proven to be effective at omitting trace, non-target eDNA from our dataset may also be the reason why we did not detect rare catches of spearfish. It is quite possible that spearfish eDNA may have been below a reasonable detection threshold and/or small water samples (150 mL per tank) did not robustly sample the whole brine tank. The DNA signal for spearfish could have been masked by more abundant species such as the tunas. It is not clear from this study what the optimal number of samples are to detect all species and more research including counts of fish presence per tank is required. Alternatively, spearfish DNA may not have been detected on some vessels because of the similarity of the 16S region in billfish (family *Istiophoridae*). Spearfish DNA was observed in vessels 6 and 7 and could have been present in samples from other vessels; however, the taxonomic assignments may have failed due to the haplotype variability in sequences making them too similar to other billfish in the 16S region. The inability to accurately assign reads to species is a common occurrence for metabarcoding research^62^ and has been identified as a shortfall in other MM studies^36,37,42^.

Species proportions were highly variable per tank across all vessels, suggesting species composition in each tank differed. All tanks were found to contain tuna spp. reads, while for some vessels the majority of billfish and pomfret reads were found in only one or two tanks (Fig. 3). This supports standard operating procedures undertaken by fishers whereby only a few tanks on the vessel are used to store billfish and pomfrets, while the rest are used for tuna (*pers comm*, industry representative). Unfortunately, the species composition in each tank was unknown for some vessels due to time and logistical constraints during unloading. Therefore, the variability of species frequency per tank is unable to be validated against logbook data and should be considered with caution. However, where tank composition was known the correct species were identified in the correct tanks. For example, vessel seven was known to contain pomfrets in tank one and all billfish in tank eight, where the majority of pomfret and billfish eDNA was identified. Obtaining better quality information at the tank level will help to estimate limits of detection (LOD) and limits of quantification (LOQ) for metabarcoding data.

The eDNA reads when organised into rank order per species were highly similar to the catch data reported in logbooks (Fig. 4). For seven of the nine vessels the eDNA reads matched the catch data when assessing most abundance to least abundant fish. In cases where rank abundance wasn’t consistant (vessel three and nine) this was due to a lack of short-billed spearfish reads being detected. Rank abundance distributions are commonly used in biodiversity modelling and allow for simple estimates of species abundance, richness, and evenness in an environment ^63^. Positive correlations between biomass, biodiversity and reads have been identified in other eDNA studies suggesting that in some situations the use of eDNA rank abundance distributions can accurately reflect fish communities^34,64,65^. Encouragingly our study has identified a similar pattern with abundance of catch able to be mirrored through ranking eDNA reads. It is of great interest if this relationship between catch abundance and reads will remain when species of tuna can be taxonomically resolved. More extended research is required to test the quantitative relationship of eDNA with catch in brine tanks especially if it is to be used as a fisheries-dependant data source.

The collection and direct preservation of water samples with Longmire’s buffer in a single tube has several benefits. The precipitation method has minimal protocol steps, requires very little equipment (gloves and tubes) and can be undertaken by non-experts. The removal of filtering equipment from the protocol reduces the chance of contamination often introduced when equipment is incorrectly sterilised, or when filter membranes are transferred for preservation^66^. The chemical properties of Longmire’s buffer, the DNA-preserving agent, also provide great benefits for the protocol and its application onboard commercial vessels. Longmire’s is highly effective at preserving eDNA at ambient temperatures removing the need for samples to be stored in refrigerators and/or freezers^44,46,67–69^. Studies have shown that eDNA preserved at ambient temperatures in Longmire’s can persist with no significant loss of DNA for 3 months^46^. Longmire’s preserved samples have also been shown to yield higher quantities of DNA when stored for short periods of time (3–8 months) due to the cell lysis efficiency of the buffer^70,71^. Our MM protocol presented in this study is proposed to be suitable for a range of fisheries stakeholders including fishers, compliance officers, fisheries scientists and NGOs all of whom may work in remote locations, have no formal genetic training and no access to refrigerators/freezers.

Molecular monitoring has the capacity to greatly increase the resolution of data collected in fisheries and our ability to trace seafood supply chains more broadly. Our study demonstrates that eDNA sampling using a simple precipitate method does well at mirroring the catch composition of a vessel. Highly abundant catch including tuna, swordfish, marlin and pomfret were accurately estimated across all vessels using eDNA. There is considerable opportunity for similar eDNA approaches to be used to better characterise by-catch and non-target species often categorised in higher-order taxa groups. For example, in the Indian Ocean Tuna Commission (IOTC) 50 per cent of all nominal shark catch is listed as ‘shark’ with no further species identification provided (IOTC2021-WPEB17DP-DATA03_NC). There have been ongoing calls to improve the collection of species-specific data on catch, discards and trade for sharks across the fishery since 2015 (IOTC-2015-S19-PropD). Enhancing the resolution of fisheries data greatly supports the management capacity including risk assessments able to be undertaken for target stocks and by-catch species^72^. Fisheries that are data-rich, well-managed and guided by formal stock assessments are in better condition than poorly managed fisheries, lacking assessments^73^. Methods for more cost-effective data collection offer significant opportunities for fisheries to advance toward a more data-rich footing, even in resource-limited contexts.

Global wild-capture fisheries are an incredibly large and diverse sector requiring creative and robust tools for effective data collection and MCS. Of course, monitoring all activity is an impossible task, however enhancing data collection and MCS capacity where possible will help to better understand what species are being captured at the fishery or regional level. Our study has demonstrated that eDNA can be used as an additional tool for collecting fisheries-dependant data for vessels with brine tanks. The eDNA sampling protocol presented here is particularly efficient at detecting abundant species retained in tanks with no detection of uncaught species. Where the protocol needs improvement is in its detection capacity for rare catches where only a few individuals may be retained in tanks. Molecular monitoring (MM) is a prominent tool already used in the fisheries sector for seafood traceability and human health. With the increasing capacity of genomic technologies and reduction in costs overtime, its likely MM will become more prominent in solving management and monitoring challenges for the fisheries sector.

### Supplementary Information

Supplementary Information.

Supplementary Tables.

## Data Availability

The datasets generated and analysed during the current study are available in the Zenodo repository; https://doi.org/10.5281/zenodo.10420788 & https://doi.org/10.5281/zenodo.10420852.
